# Anti-proliferative effect of novel primary cetyl alcohol derived sophorolipids against human cervical cancer cells HeLa

**DOI:** 10.1371/journal.pone.0174241

**Published:** 2017-04-18

**Authors:** Laxman Nawale, Parul Dubey, Bhushan Chaudhari, Dhiman Sarkar, Asmita Prabhune

**Affiliations:** 1 Combichem-Bioresource Center, OCD, National Chemical Laboratory, Pune, India; 2 Biochemical Sciences Division, National Chemical Laboratory, Pune, India; Institute of Biochemistry and Biotechnology, TAIWAN

## Abstract

Sophorolipids (SLs) are glycolipid biosurfactants that have been shown to display anticancer activity. In the present study, we report anti-proliferative studies on purified forms of novel SLs synthesized using cetyl alcohol as the substrate (referred as SLCA) and their anticancer mechanism in human cervical cancer cells. Antiproliferative effect of column purified SLCA fractions (A, B, C, D, E and F) was examined in panel of human cancer cell lines as well as primary cells. Among these fractions, SLCA B and C significantly inhibited the survival of HeLa and HCT 116 cells without affecting the viability of normal human umbilical vein endothelial cells (HUVEC). The two fractions were identified as cetyl alcohol sophorolipids with non-hydroxylated tail differing in the degree of acetylation on sophorose head group. At an IC_50_ concentration SLCA B (16.32 μg ml^-1^) and SLCA C (14.14 μg ml^-1^) blocked the cell cycle progression of HeLa cells at G1/S phase in time-dependent manner. Moreover, SLCA B and SLCA C induced apoptosis in HeLa cells through an increase in intracellular Ca^2+^ leading to depolarization of mitochondrial membrane potential and increase in the caspase-3, -8 and -9 activity. All these findings suggest that these SLCAs could be explored for their chemopreventive potential in cervical cancer.

## Introduction

Sophorolipids (SLs), belong to the class of glycolipid biosurfactants that are synthesized extracellularly by certain non-pathogenic yeasts. SLs initially gained attention because of the alkane utilizing ability of the yeasts. But later they exponentially attained recognition owing to possession of several properties such as emulsification, anti-microbial, anti-viral, anti-cancer that played role in various fields like detergent industry, cosmetics, pharmaceuticals etc. [1].

Anti-cancer property of SLs has been extensively studied in past owing to their promising potential and biocompatibility. Researchers have elucidated the cytotoxic effects of SLs produced from *Wickerhamiella domercqiae* against human lung cancer A549, liver cancer H7402 and esophageal cancer KYSE109, KYSE450 respectively [2,3]. The antiproliferative activity of SL against H7402 liver cells was accounted to its apoptosis- inducing ability marked by morphological changes such as cell shrinkage, chromatin condensation and membrane blebbing [4]. Enhanced cytotoxic effect of SLs obtained from *Candida bombicola* against human pancreatic carcinoma cells was demonstrated by their selective derivatization into alkyl esters [5]. Promising anticancer activity of SLs against hepatocellular carcinoma HepG2 and lung adenocarcinoma A549 due to inhibition of urokinase and histone deacetylase activities has also been reported [6].

Structurally, classical sophorolipids comprise of a hydrophilic dimeric sugar head group known as sophorose, linked glycosidically to a hydrophobic tail of 16–18 carbon fatty acids. But, structure-bioactivity relationship of SLs has been tested with a view to achieve enhanced properties by varying the lipophilic feed of the yeast ranging from alkane, fatty acid to fatty alcohol. Similarly, to achieve superior biological properties, chemoenzymatic modification of SLs has also been done [7]. Sophorolipid synthesis has opened new facet for direct applicability and employment of several hydrophobic molecules which being water insoluble have limited biological applications or other setbacks.

Microbial conversion of similar water insoluble lipophilic substrate, cetyl alcohol, also commonly known as palmityl alcohol [CH_3_(CH_2_)_14_CH_2_OH], into amphiphilic sophorolipid molecule was carried out as reported previously [8]. Prior presence of hydroxyl group in the fatty alcohol probably bypasses the hydroxylation step in the biosynthetic pathway of SLs. Thus, altered SLs differing in the hydrophobic tail end with—CH_3_ and—CH_2_OH groups are synthesized as a mixture. This modification from the classical SLs (C18, acidic and lactonic) is expected to impart enhanced or suppressed biological properties comparatively.

Since glycolipids have been shown to possess anticancer activity, novel SLs synthesized using cetyl alcohol were subjected to purification using silica gel chromatography and purified fractions were studied for their toxicity against different human cancer cell lines: acute monocytic leukemia THP-1, cervical carcinoma HeLa, colon carcinoma HCT 116, lung adenocarcinoma A549, breast adenocarcinoma MCF-7, pancreas carcinoma PANC-1, and squamous carcinoma A431. Further, the underlying mechanism of anti-proliferative behaviour of SLCAs was studied on HeLa.

## Materials and methods

### Sophorolipid production and column purification

The yeast *Candida bombicola* ATCC 22214 was used for sophorolipid production following the procedure as described previously [8] (Refer supplementary information). The crude sophorolipid obtained by fermentation was separated by silica gel column chromatography. Brown viscous SLCA was chromatographed on a silica gel column (100–200 mesh size). Elution was performed using chloroform/methanol with increasing amount of methanol (99:1, 98:2 upto 95:5). Successive fractions were collected at regular time interval and solvent was dried under vacuum by rota evaporation. The purity of the compound was primarily checked by thin layer chromatography using chloroform/methanol in 9:1 ratio as solvent. Finally, liquid chromatography mass spectroscopy (LC-MS) was carried out for confirming the structure of the purified sophorolipid by comparing with already reported data [8]. Sophorolipids were then dissolved in sterilized Mili Q water at 2mg/mL concentration and diluted to working range (10–320 μg ml^-1^).

### Cell lines and cell culture

THP-1, HeLa, HCT 116, MCF-7, A549, PANC-1 and A431 cell lines were procured from National Centre for Cell Science (NCCS), Cell Repository, Pune. The procured cell lines were approved by NCCS ethics committee. HUVEC primary cells were obtained from Invitrogen.

THP-1 cells were maintained in RPMI 1640; HeLa and MCF-7 cells in Eagle's Minimum Essential Medium (EMEM); L929, A549 and PANC-1 cells in Dulbecco’s Modified Eagle Medium (DMEM) supplemented with 10% fetal bovine serum (FBS). HUVEC cells were cultured in M200 media enriched with 50X large vessel endothelial supplement (LVES) (Gibco, Invitrogen). All the cells were incubated in 5% CO_2_ at 37°C.

### Cell viability

The cell viability was determined by MTT dye uptake as described previously [9]. Briefly, 1x10^5^ cells ml^-1^ were seeded in 96-well plate. Next day, cells were treated with increasing concentrations of sophorolipids (10, 20, 40, 80, 160, 200, 250, 320 μg ml^-1^ final concentrations) in sterilised MiliQ water and incubated at 37°C with 5% CO_2_ for 48 h. An untreated group was kept as a negative control and cells treated with doxorubicin (0–10 μg ml^-1^) or paclitaxel (0–12.5 μg ml^-1^) were used as positive controls. Wells containing culture medium and MTT but no cells acted as blanks. As same solvent (Mili Q water) has been used for control and blank additional experiment was not conducted. After incubation, the MTT solution (5 mg ml^-1^) was added to each well and the cells were cultured for another 3 h at 37°C in 5% CO_2_ incubator [10]. The formazan crystals formed were dissolved by addition of 200 μl of 0.04N acidified isopropanol. After 15 min, the amount of colored formazan derivative was determined by measuring optical density (OD) using the microplate reader at 570 nm. The percentage inhibition was calculated as: % Inhibition = [(OD of control well—OD of treated well)/ (OD of control well—OD of blank)] × 100. All experiments were conducted in triplicates and respective measurements were presented as the average ± standard deviation.

### Cell cycle distribution

The distribution of sophorolipid treated cells in cell cycle was assayed as described earlier [11] by DAPI staining at different time intervals. Briefly, HeLa cells were treated with sophorolipids SLCA B (16.32 μg ml^-1^) and SLCA C (14.14 μg ml^-1^) for time interval of 6, 12, 18 and 24 h. Following incubation, cells were stained with DAPI and the DNA content was measured using a laser-scanning confocal microscope (LSCM). Data was analysed using Thermo Scientific^™^ HCS studio^™^ 2.0 software to calculate the percentages of cells in G1, S and G2/M phase.

### Apoptosis by annexin V-FITC PI staining

Apoptosis was evaluated by the binding of annexin V-FITC to phosphotidylserine, that gets externalized to the outer leaflet of the plasma membrane [12], followed by high content screening. Post-incubation, the cells were harvested and subsequently treated with annexin V-binding buffer comprising of annexin V-FITC (3 μg ml^-1^), DAPI (1 μM) and propidium iodide (10 μg ml^-1^) [13]. The number of cells undergoing apoptosis were examined using LSCM (20X magnification, Olympus FV1000) and Thermo Scientific^™^ HCS studio^™^ 2.0 software was used for three dimensional multichannel-image processing. The apoptotic ratio was calculated as: % apoptotic ratio = [(the number of cells positive for Annexin V-FITC)/ (the number of cells positive for DAPI)] ×100

### Mitochondrial membrane depolarization (Δψm)

Mitochondrial membrane potential (MMP) was investigated using Mito Tracker Red (Invitrogen), a mitochondrial potential sensor [14]. Briefly, HeLa 1x10^5^ ml^-1^ cells treated with sophorolipids SLCA B (16.32 μg ml^-1^) and SLCA C (14.14 μg ml^-1^) for 2, 24, 48 and 72 h were stained with 0.1 μmol l^-1^ Mito Tracker Red and incubated at 37°C for 15 mins. After incubation, the cells were washed, fixed and then stained with DAPI (1 μM ml^-1^). Further, LSCM was used to acquire images of the stained cells by employing HCS using Mito Tracker Red (a red-fluorescent dye) at excitation wavelength 525 nm and emission wavelength 590 nm. Analysis was carried out using Thermo Scientific^™^ HCS studio^™^ 2.0 software.

### Intracellular cytoplasmic calcium release [Ca^2+^]i

The effect of SLCAs on [Ca^2+^]i release was examined quantitatively and qualitatively using Fluo 4-acetoxymethyl ester (Fluo-4 AM) dye as described previously [15]. Briefly, HeLa 1x10^5^ ml^-1^ cells were treated with sophorolipids for 4, 8 and 12 h. Following incubation, the cells were washed with PBS and loaded with Fluo-4 AM (4 μM). Nuclei were stained with DAPI. An alteration in released calcium level was detected using LSCM by measuring the green fluorescence (excitation 490 nm, emission 530 nm). Data analysis was carried out using Thermo Scientific^™^ HCS studio^™^ 2.0 software.

### Caspase activity

Caspase activities were quantified using Caspase Activity Assay Kit (Promega, USA) according to the manufacturer’s instructions and as described previously. Apo Alert Caspase Luminescent Assay Kit (Promega, USA) and EnzChek Caspase-3 Assay Kit (Molecular probes, USA) were used for measuring caspase -8, -9 and -3 activity, respectively. Briefly, HeLa cells were treated with SLCAs and incubated for indicated time periods. The cells were lysed, centrifuged and supernatant were mixed with equal volumes of 2X reaction buffer along with specific substrate conjugate: (Z-DEVD—AMC) for caspase-3, acetyl-Ile-Glu-Thr-Asp p-nitroaniline (Ac-IETD-pNA) for caspase-8 and acetyl-Leu-Glu-His-Asp-p-nitroaniline (Ac-LEHD-p-NA) for caspase-9 and incubated at 37°C for 1 h. The resultant fluorescence intensity of the cleaved product after incubation was quantified using plate reader (VarioskanFlash, using SkanIt Software 2.4.5 RE, Thermo Scientific) with 496/520-nm filters for caspase-3, 400/500-nm filters for caspase-8 and 380/460-nm filters for caspase-9. The increase in caspase activity was calculated for sophorolipid treated cells with respect to the control cells.

### Statistical analysis

All the experiments were performed in triplicates and repeated at least twice and the data has been presented as mean ± SD. Statistical analysis was conducted with the SigmaStat 3.5 program (Systat Software, Inc.) using one-way ANOVA and the associated p values have been noted.

## Results

### Cetyl alcohol sophorolipid

Cetyl alcohol sophorolipid was obtained as a brown viscous product by solvent extraction procedure. The synthesized product chromatographically revealed to be a mixture of different forms. With the help of column chromatography we could successfully isolate 6 fractions (A-F). The two sophorolipids that showed promising biological activity in this study were confirmed to be cetyl alcohol derived sophorolipids. The significant ions that occurred at m/z 589 and m/z 631 for fractions B and C were sodium adducts (spectra and structure provided as S1 and S2 Figs). The structures were determined as the non-acetylated and mono-acetylated forms of sophorolipid with sophorose head group and 16 carbon fatty tail ending with terminal methyl group, respectively. These two compounds were further abbreviated as SLCA B and SLCA C for cell studies.

### SLCAs inhibits growth of HeLa and HCT 116 cells

**Fig 1:**
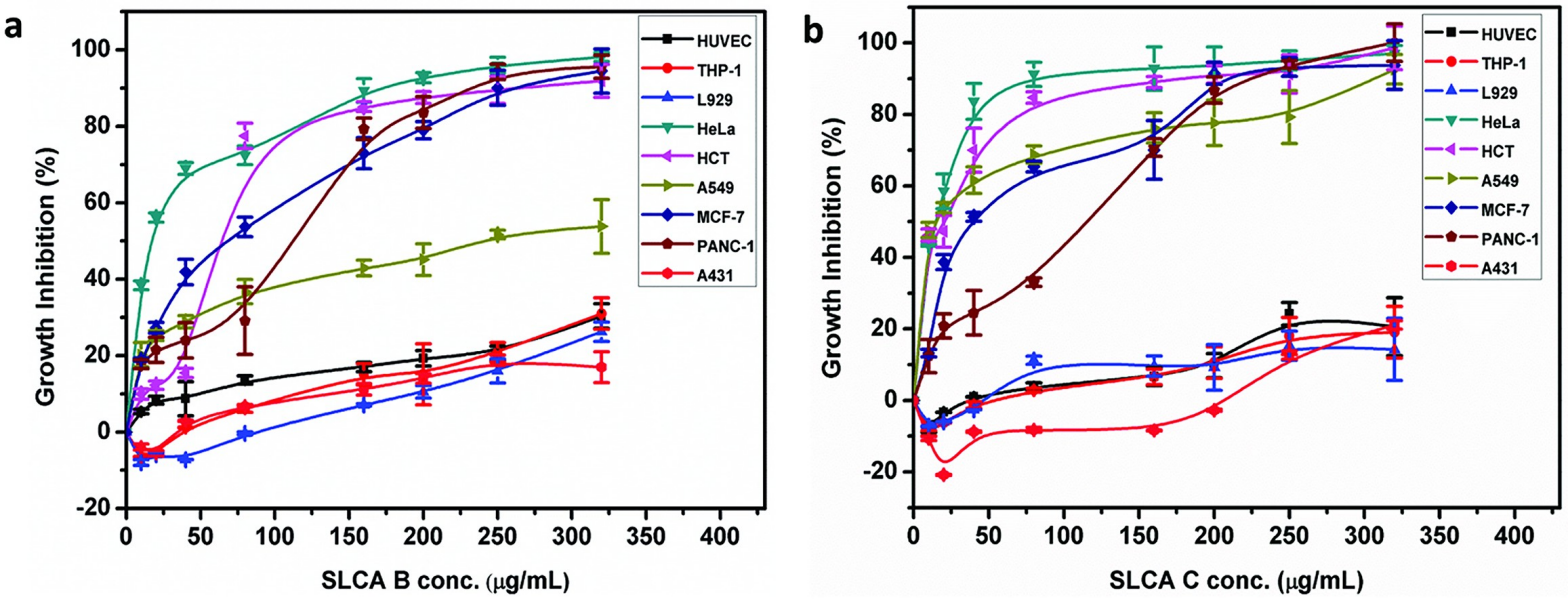
SLCAs inhibits growth of cancer cells. Cancer cell lines as well as primary HUVEC cells were subjected to different concentrations of SLCA B, SLCA C, doxorubicin and Paclitaxel for 48 h. Graphical representation of percent growth inhibition GI_50_ for (a) SLCA B (b) SLCA C. The data represents mean ± SD of three independent experiments.

To examine the antitumor activity of SLCA (A, B, C, D, E, F) fractions, human cancer cells (THP-1, HeLa, HCT 116, A549, MCF-7, PANC-1 and A431) as well as exponentially dividing primary (HUVEC) cells were treated with increasing concentrations of SLCAs, and cell viability was measured over time by the MTT assay (Table 1, Fig 1a and 1b). Doxorubicin and paclitaxel were used as reference standard. It was observed that among all the fractions, SLCA B and SLCA C significantly inhibited the growth and decreased the viability of HeLa (GI_50_; SLCA B: 16.32 μg ml^-1^, SLCA C: 14.14 μg ml^-1^ and GI_90_; SLCA B: 162.08 μg ml^-1^, SLCA C: 71.47 μg ml^-1^) and HCT 116 (GI_50_; SLCA B: 62.77 μg ml^-1^; SLCA C: 23.22 μg ml^-1^ and GI_90_; SLCA B: 262.9 μg ml^-1^; SLCA C: 170.68 μg ml^-1^) cells in a concentration-dependent fashion (Fig 1a). Moreover, SLCA B and SLCA C had GI_50_ value above 65 μg ml^-1^ and 14 μg ml^-1^, respectively, in A549, MCF-7, PANC-1 and A431 cells (Table 1). Interestingly, both SLCA B and SLCA C did not demonstrate the toxicity towards primary HUVECs and on L929 mouse fibroblasts (<20% at 320 μg ml^-1^). These preliminary data suggested that both the SLCA B (Fig 1a) and SLCA C (Fig 1b) had high inhibitory effect on human cervix adenocarcinoma (HeLa) and colon carcinoma (HCT 116) cells, with significant activity in HeLa cells even at lower concentration. In order to understand the mechanism by which SLs functioned, we further investigated the effects of SLCA B and SLCA C in HeLa cells.

**Table 1:** GI_50_ value of treatment on primary as well as cancer cell lines.

Cell Name	SLCA A (μg ml^-1^)	SLCA B (μg ml^-1^)	SLCA C (μg ml^-1^)	SLCA D (μg ml^-1^)	SLCA E (μg ml^-1^)	SLCA F (μg ml^-1^)	^a^Doxorubicin (μg ml^-1^)	^a^Paclitaxel (μg ml^-1^)
**HUVEC**	> 320	> 320	> 320	> 320	> 320	> 320	>10	>10
**THP-1**	> 320	> 320	> 320	> 320	> 320	> 320	>10	0.14±0.01
**L929**	> 320	> 320	> 320	> 320	> 320	> 320	>10	>10
**HeLa**	191.87±2.31	**16.32±3.81**	**14.14±3.76**	> 320	164.5±3.64	> 320	1.45±0.45	0.005±0.00
**HCT 116**	64.56±5.2	**62.77±12.25**	**23.22±2.89**	> 320	103.08±4.29	> 320	0.54±0.01	0.026±0.01
**A549**	190.31±5.78	236.32±6.39	14.95±3.52	> 320	>320	> 320	0.87±0.19	0.004±0.001
**MCF -7**	239.76±6.36	65.7±3.38	37.71±5.44	248.93	255.15±3.81	256.86±6.86	2.52±0.43	0.002±
**PANC-1**	> 320	113.52±4.66	116.33±5.35	> 320	> 320	> 320	3.96±0.76	0.128±0.01
**A431**	> 320	> 320	> 320	> 320	> 320	> 320	9.05±0.90	1.64±0.39

^a^ Standard anticancer drug as a positive control.

The data represents mean ± SD of three independent experiments.

### SLCAs induce G1/S phase arrest in HeLa cells

**Fig 2:**
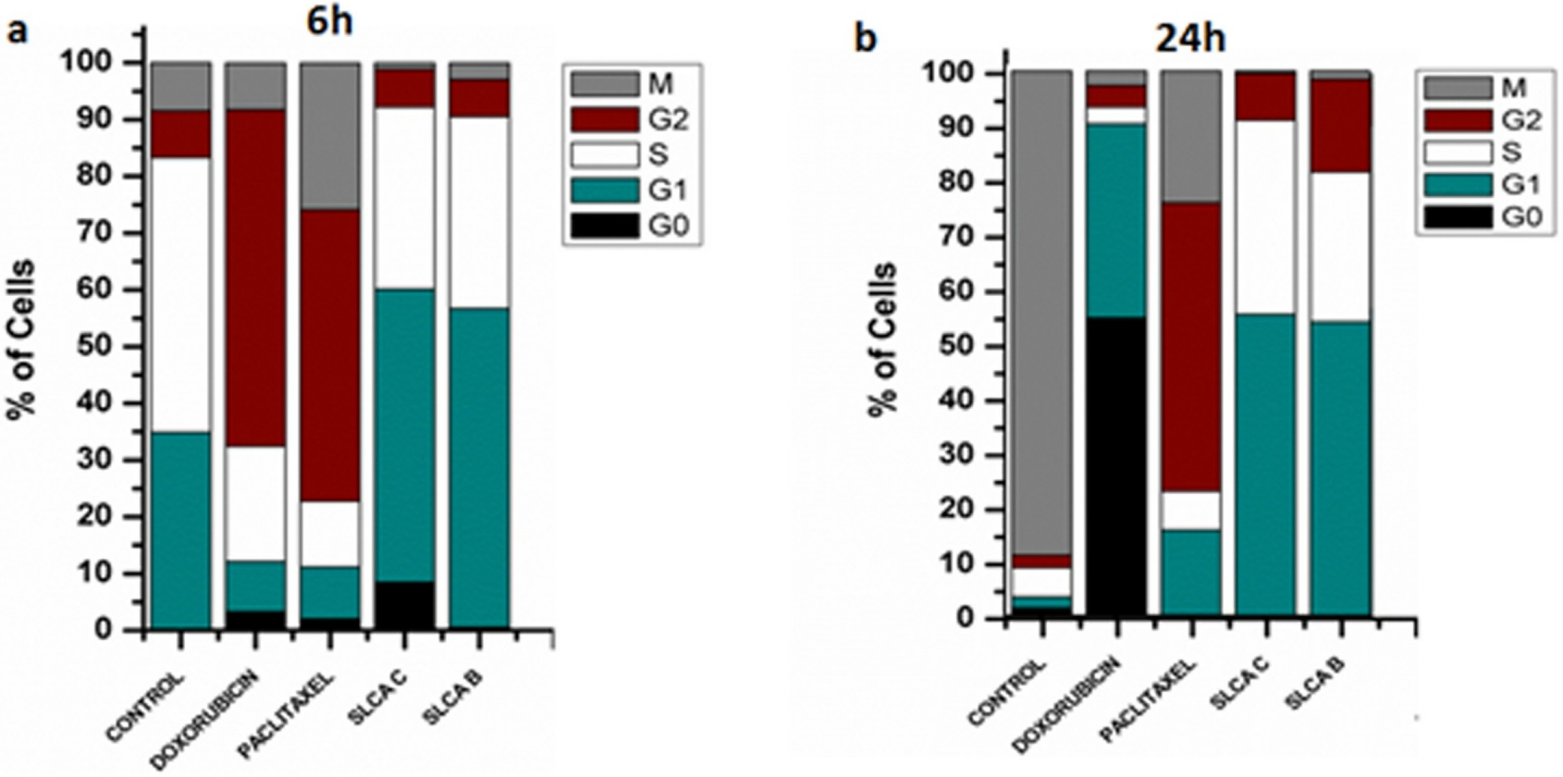
SLCAs induce G1/S phase arrest in HeLa cells. HeLa cells were subjected to different treatment at GI_50_ concentration for different time interval (6, 12, 18 and 14h). Post treatment, cells were monitored with DAPI staining and were analyzed by HCS. Graphical representation of % cells in different phases of cell cycle at 6h (a) and 24h (b).

To analyse the mechanism behind SLCAs-induced growth inhibition in cervical cancer cells, we investigated the cell cycle distribution in HeLa cells (Table 2, Fig 2a and 2b). HCS-DAPI staining analysis showed that in presence of SLCA for 24 h, HeLa cells exhibited an increase in the G1 (SLCA B: 53.94%; SLCA C: 55.24%) and S (SLCA B: 27.45%; SLCA C: 35.52%) phase with a decrease in M (SLCA B: 1.52%; SLCA C: 0.45%) phase population as compared to untreated (G1: 7.13%; S: 2.2%; M: 88.74%) cells, indicative of early G1-S block. Moreover, doxorubicin treatment (6 h) resulted in G2 (59.33%) phase arrest whereas further continuous treatment for 24 h resulted in arrest at G_0_/G_1_ phase, whereas at similar time point paclitaxel treatment resulted in G2/M phase (52.87% / 24.17%) arrest.

**Table 2:** Percent of cells in different phase of cell cycle at different time interval in treated (DOX: Doxorubicin, PATA: Paclitaxel, SLCA B, SLCA C) and untreated (CNT-Control) cells.

Time	Treatment	G0	G1	S	G2	M
**6 h**	CNT	0	34.91±3.95	48.42±0.08	8.22±4.67	8.45±0.81
	DOX	3.28±1.26	8.87±0.04	20.27±3.17	59.33±5.10	8.24±3.15
	PATA	2.05±1.97	9.12±0.86	11.57±2.28	51.39±3.78	25.86±2.60
	SLCA C	8.47±2.20	**51.67±0.55**	32.06±0.83	6.66±0.72	1.13±0.10
	SLCA B	0.52±0.21	**56.21±4.99**	33.81±3.58	6.55±0.23	2.89±1.38
**12h**	CNT	4.97±0.00	7.13±0.30	6.86±0.02	6.88±0.11	74.15±0.16
	DOX	**54.92±3.59**	37.34±0.76	2.37±0.03	5.54±2.45	2.82±2.37
	PATA	1.78±0.14	9.46±0.33	14.52±2.01	**45.73±3.92**	28.50±1.73
	SLCA C	4.21±3.90	**54.33±2.02**	32.72±0.90	7.7±0.62	1.03±0.35
	SLCA B	2.71±1.54	**52.30±0.60**	31.71±2.40	9.31±1.31	3.96±3.24
**18h**	CNT	1.63±0.35	4.68±0.01	4.66±0.01	4.74±0.10	84.29±0.25
	DOX	**54.70±2.44**	34.98±0.91	2.69±0.51	4.91±0.61	2.71±2.23
	PATA	2.43±0.33	10.85±1.15	12.02±1.82	**52.21±2.52**	22.47±0.12
	SLCA C	3.49±0.15	**55.33±2.20**	31.14±3.04	9.17±1.91	0.86±0.92
	SLCA B	5.69±5.28	**53.21±1.68**	32.27±0.88	7.50±2.61	1.32±0.11
**24h**	CNT	1.565±0.35	2.02±0.25	5.34±0.62	2.32±0.02	88.74±0.49
	DOX	**54.70±3.20**	35.61±4.55	3.03±1.35	4.01±4.03	2.63±2.37
	PATA	0.29±0.28	15.55±1.28	7.10±1.21	**52.87±2.55**	24.17±2.34
	SLCA C	0.17±0.25	**55.24±0.21**	35.52±0.80	8.60±0.35	0.45±0.49
	SLCA B	0.09±0.00	**53.94±2.01**	27.45±1.71	16.99±4.43	1.52±0.71

The darkened numbers indicate the percentage of cells arrested in different phases of cell cycle.

The data is mean ± SD of three independent experiments.

### Induction of apoptosis in HeLa cells by SLCAs

**Fig 3:**
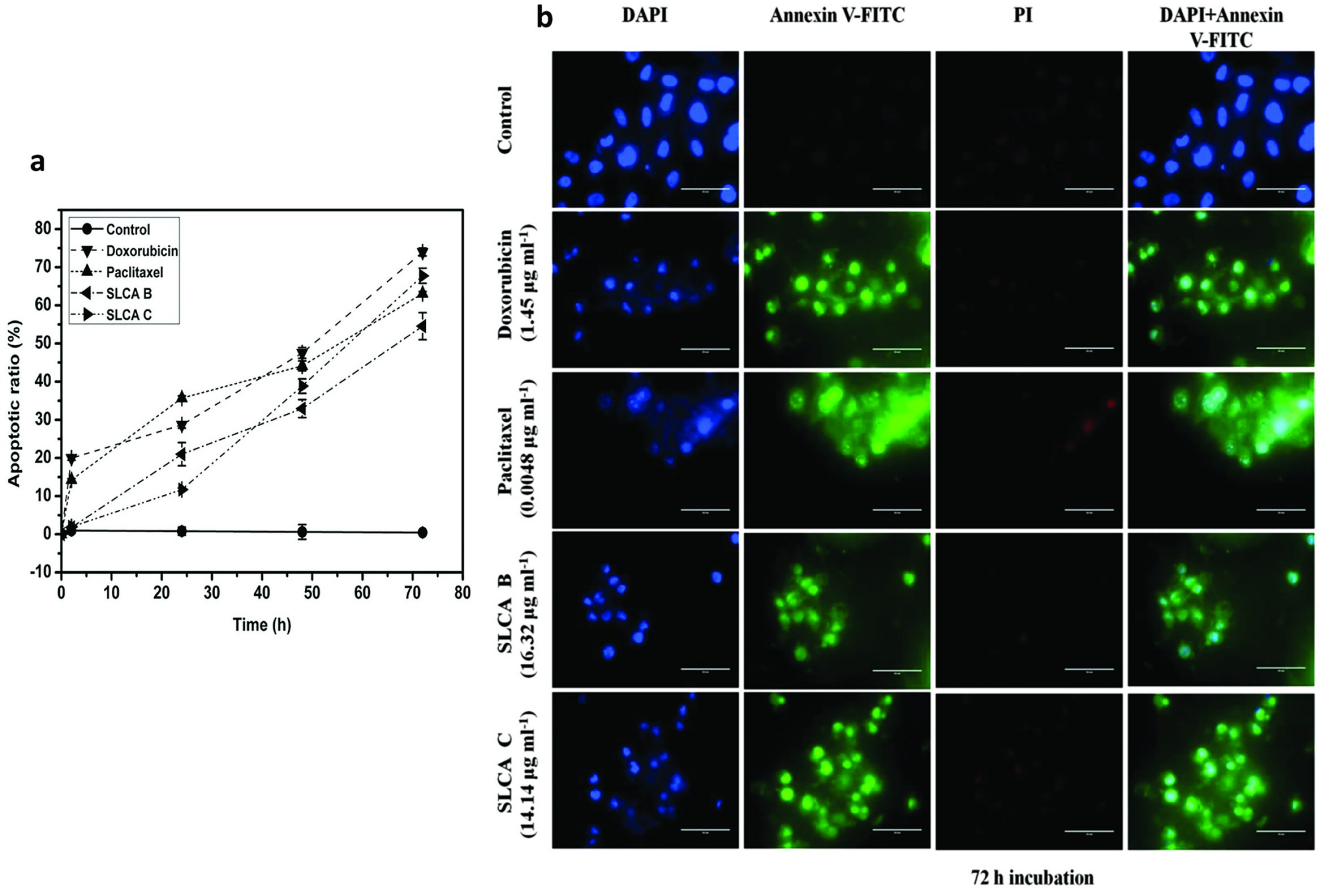
SLCAs induce apoptosis in HeLa. (a) Apoptotic ratio (b) Annexin-V (AV) and PI labeling of HeLa cells counterstained with DAPI. The overlay images in the second last and last column represent the apoptotic cells and absence of necrosis respectively. The analysis was conducted using confocal microscopy, Magnification 20X (scale, 100 μm).

To further elucidate whether SLCAs mediated decrease in cell growth was due to apoptosis, we investigated apoptosis in HeLa cells by SLCA B and SLCA C using annexin V-FITC/PI and DAPI as staining agents. It was observed that treatment of HeLa cells with GI_50_ concentration (SLCA B: 16.32 μg ml^-1^; SLCA C: 14.14 μg ml^-1^) resulted in gradual increase of apoptotic cells in a time-dependent manner (Fig 3a and 3b). The calculated average apoptotic ratios upon 72 h for SLCA B and SLCA C treatment were 67.75 ±9.17 (p = 0.006) and 54.2 ±3.55 (p = 0.017) respectively as compared to untreated control 0.46±0.52. At similar time point, doxorubicin and paclitaxel showed apoptotic ratios 73.97±1.02 (p = 0.002) and 63.11±0.49 (p = 0.001) respectively. Moreover, PI-positive cells were not detected in the entire experimental time frame (72 h) suggesting absence of necrosis (Fig 3b). This indicated the potential of SLCA B and SLCA C to induce cell death *via* apoptotic pathway in HeLa cells.

### Decrease in mitochondrial membrane potential and increase in intracellular calcium in HeLa cells by SLCAs

**Fig 4:**
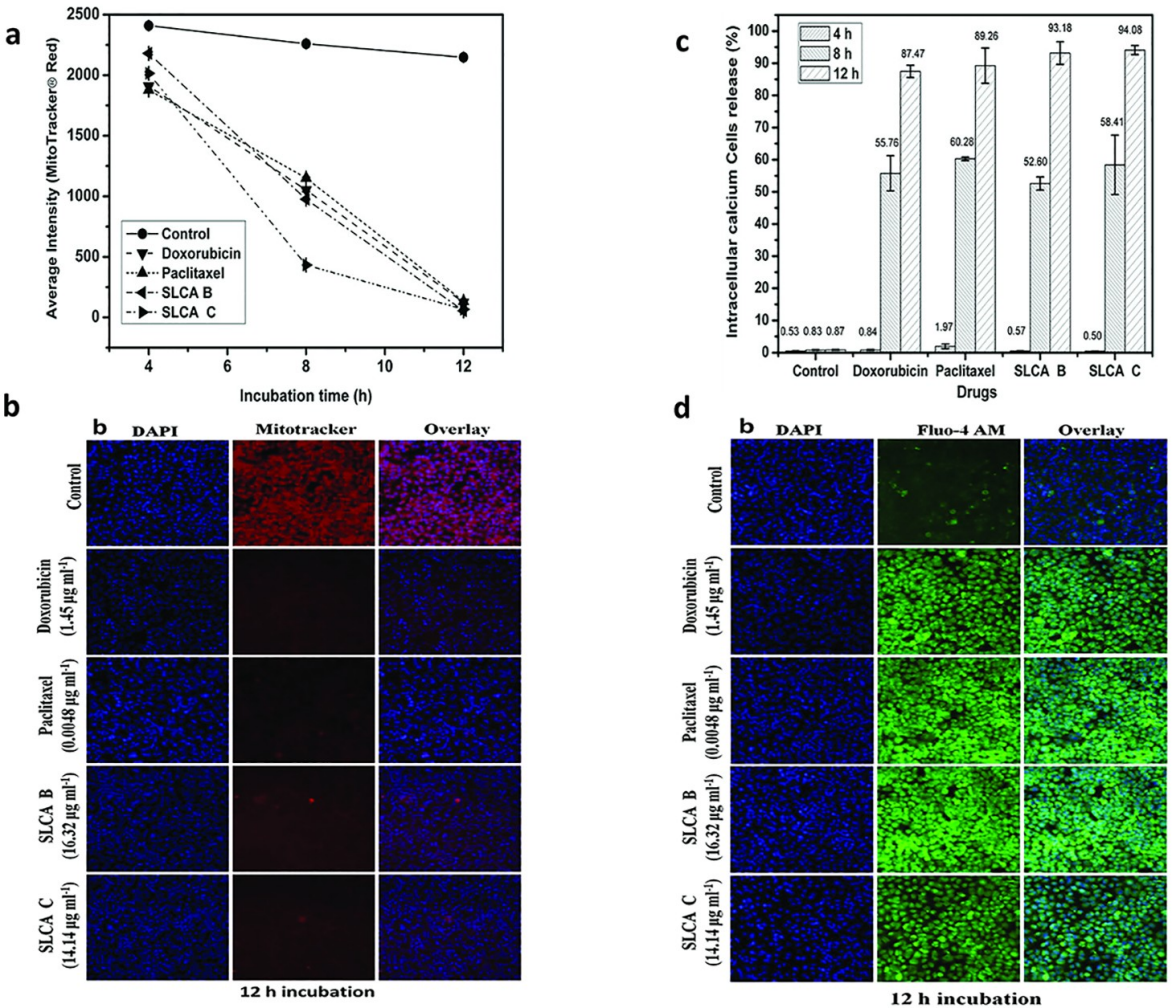
SLCAs decrease mitochondrial membrane potential and increase intracellular calcium in HeLa cells. Alteration of mitochondrial transmembrane potential in sophorolipid treated HeLa cells as measured by staining with Mito Tracker Red. (a) Average dye intensity signifying depolarized cells. Data from triplicate experiments represented as mean±SD (b) Fluorescence intensity of bound dye as recorded by confocal microscope (20X magnification, scale-100 μm). Elevation in intracellular [Ca^2+^]i on sophorolipid treatment of HeLa cells as measured by Fluo-4AM. (c) Bar diagram represents cell percentage releasing calcium at different time intervals. (d) Overlay of confocal microscopy images exemplifying increased Fluo-4 AM intensity after 12 h that indicates increase in cytoplasmic calcium. Magnification 20X (scale, 100 μm).

To test whether the SLCA-induced apoptosis in the HeLa cells is associated with mitochondrial dysfunction, we determined mitochondrial membrane potential, following staining with Mito Tracker Red dye. As shown in Fig 4a and 4b, the intensity of red fluorescence decreased significantly within 8 h after sophorolipid-treatment and diminished markedly thereafter (Fig 4a). This clearly indicated that SLCAs caused mitochondrial membrane depolarization evident from Fig 4b exemplifying loss in red intensity at 12 h.

Since decrease in mitochondrial membrane potential is known to increase intracellular Ca^2+^, we measured the levels of intracellular Ca^2+^ in HeLa cells treated with SLCAs followed by staining with calcium sensitive dye Fluo-4 AM. The results showed that the cells treated with SLCA B (16.32 μg ml^-1^) and SLCA C (14.14 μg ml^-1^) displayed an increase in the green fluorescence than the control cells in time-dependent manner (Fig 4c and 4d). Interestingly, HeLa cells treated with SLCA B, SLCA C, doxorubicin and paclitaxel for 12 h showed 93.2% (p = 0.017), 94.1% (p = 0.007), 87.4% (p = 0.009), and 89.2% (p = 0.028) increase in calcium ions as compared to the control (0.87%).

### Caspase-dependent cell death in HeLa cells by SLCAs

**Fig 5:**
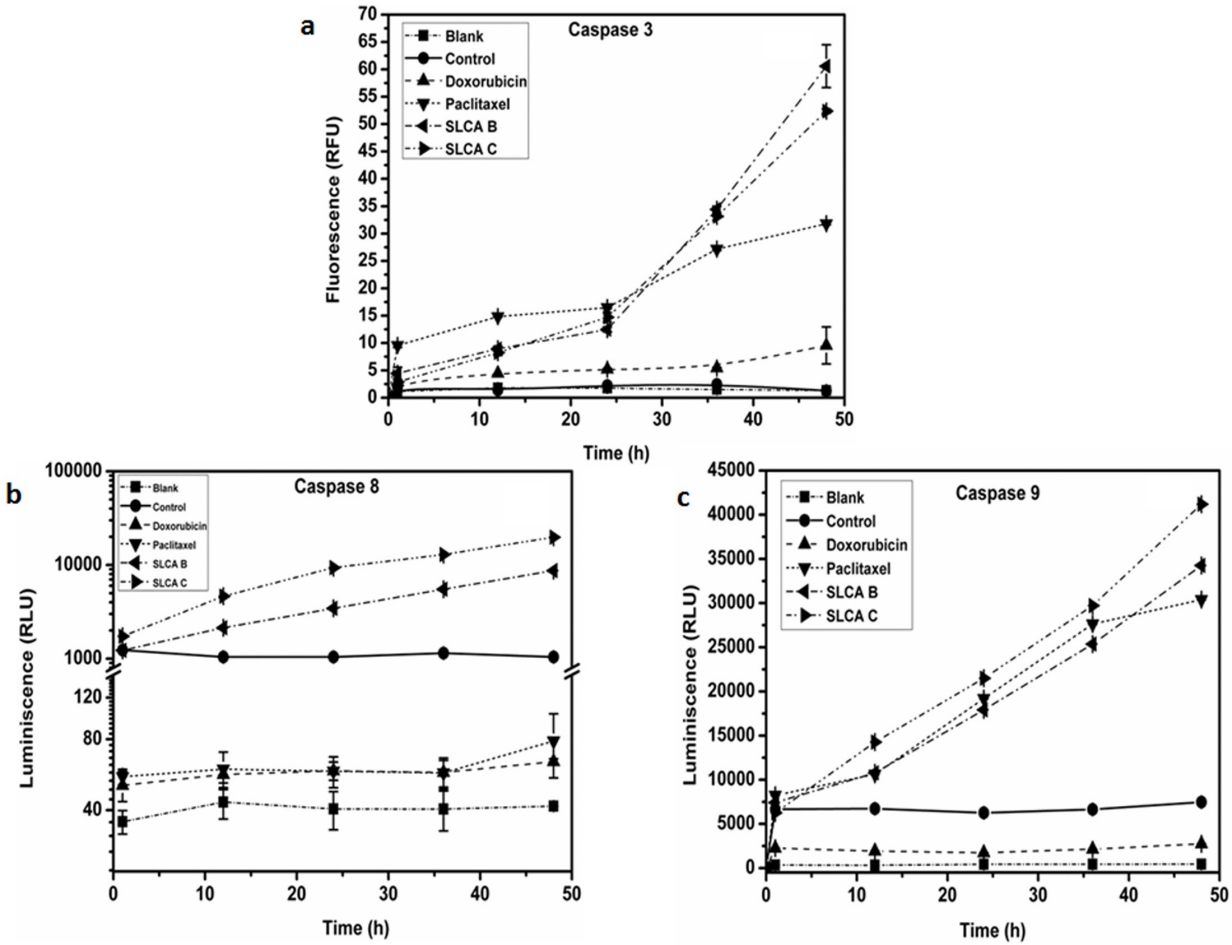
Effect of SLCA B and SLCA C on activation of caspases. The sophorolipid treated cells were incubated for different time intervals and caspase activity was assessed by spectrometric method. (a) caspase-3, (b) caspase-8 and (c) caspase-9 activity was measured in comparison to the control. Data represented mean ± SD values.

The apoptotic process primarily includes the activation of cysteine proteases, which represent both initiators and executors of cell death [16]. To investigate whether induction of apoptosis by SLCA involves the activation of caspase-aspartate-specific cysteine proteases, we measured the procaspases-8 and -9 processing associated with the apoptotic pathways. SLCA B (16.32 μg ml^-1^) and SLCA C (14.14 μg ml^-1^) enhanced caspase-3, 8 and 9 expressions strongly in a time-dependent manner in HeLa cells when compared to the untreated cells (Fig 5a, 5b and 5c respectively). At 24 h upon SLCA B treatment, there was ~5.5 (p = 0.001), 3.3 (p = 0.001) and 2.9 (p = 0.023) -fold increase in activity of caspase-3, -8 and -9 respectively. Similarly, SLCA C treatment showed ~6.5 (p = 0.019), 9.0 (p = 0.035) and 3.4 (p = 0.001)—fold increase in activity of caspase-3, -8 and -9 respectively. The enzyme activity was reduced to negligible level in the presence of inhibitors. On the other hand, pre-treatment of caspase-8 inhibitor, inhibits caspase-9 activation. These results suggest that both SLCAs induce cell death *via* pro-apoptotic molecules in HeLa cancer cells.

## Discussion

Sophorolipids (SLs) are glycolipid molecules that are considered to be of great interest in management of various ailments owing to their varied biological properties. SLs being amphiphilic in nature partially resemble the cell membrane lipids, thus they are able to span through the membrane and gain entry inside the cell. Additionally, the ability of SLs to selectively affect cancerous cells without harming normal cells may be considered beneficial in minimizing the side effects that are generally associated with current therapeutic regimens [7]. SLs have shown to possess anticancer effect against various cancers such as pancreatic, liver and lung. Recently, *in vitro* and *in vivo* anticancer activity of di-acetylated lactonic SLs of varying chain length and degree of unsaturation has been reported in human cervical cancer [17]. Moreover, we have recently reported the anti-cancer activity of crude cetyl alcohol and oleic acid SLs in breast and cervical cancer cell lines [18]. The SLs described in the reported work comprise of a mixture of structurally different forms varying in degree of acetylation on sophorose, fatty acid chain length and internal esterification of end carboxylic acid group (in oleic acid SL). After confirming the presence of anti-cancer activity in the crude SLCAs it becomes necessary to investigate and report the activity for the purified forms (Refer S3 Fig). Prior presence of extensive literature on anti-proliferative property of oleic acid SL [2,4,5,19] prompted us to divert the investigation on unexplored purified forms of novel cetyl alcohol SLs synthesized microbially by *Candida bombicola*. Thus, the present study was extended by initially conducting cytotoxicity study of six different column purified fractions of cetyl alcohol sophorolipids (SLCA A, B, C, D, E, F) on panel of cancer cell lines and primary cells. The results revealed higher potency of SLCAs B and C against cervical cancer cells (HeLa) without affecting the viability of normal cells. The superior growth inhibition activity of both SLCA B and SLCA C in comparison to other SLCA could be attributed to difference in their structure with others. Using LC-MS, the fractions SLCA B and SLCA C were identified as the non-acetylated and mono-acetylated SL molecules respectively with sophorose head group conjugated to 16 carbon non-hydroxylated fatty tail (Refer S1 and S2 Figs). The methylated end in SLCA B and presence of additional acetyl group in SLCA C increases the hydrophobicity of the molecule. Similarly, studies on anti-cancer efficacy of lactonic sophorolipids against esophageal cancer cells also show that more hydrophobic diacetylated lactonic SL is more effective than the monoacetylated lactonic SL [3]. Thus, in the case of cetyl alcohol SL we can hypothesize that SLCA B & C being more hydrophobic in comparison to other SLCA forms display superior growth inhibition activity.

Additionally, the selective strategy of sophorolipids towards killing cancer cell and not normal cell (in concentration range studied) could be attributed to the increased susceptibility of cancer cells due to their rapidly dividing nature. Also the impaired ability of cancer cells to repair any DNA damage, subjects them to apoptosis [20]. Thus, probably SLCAs act on tumor cells at fairly low concentration whereas at higher concentrations also they do not harshly impact the normal cells whose repair mechanism is intact. We further elucidated the anti-neoplastic potential of these two SLCAs in cervical cancer cells with the possible underlying mechanisms.

Physiological processes such as proper tissue development and homeostasis, essentially require a balance between cell proliferation and apoptosis that are linked by cell-cycle regulators and apoptotic stimuli affecting both the processes [21]. SLCAs inhibited the cell proliferation in human cervix cancer cells which was associated with cell cycle arrest in G1 phase in HeLa cells. In HPV positive cervical cancer cells deregulation of G1/S phase transition is a common phenomenon, which reduces the G1 phase and accelerates the G1/S transition [22]. The treatment of HeLa cells with SLCAs arrests the G1 phase indicating the potential of SLCAs to control the transition of cells from G1 to S phase. HeLa cells contain HPV-18 genome (10–50 copies/cell) along with the host genome. The viral genome codes for two oncoproteins, E6 and E7 which initiates degradation and destabilization of tumor suppressor proteins, p53 and pRB, respectively. The degradation of p53 leads to cell proliferation and inhibition of apoptosis; whereas destabilization of pRB leads to deregulation of the cell cycle [23]. Thus, in cetyl alcohol SL treated HeLa cells; we hypothesize that both SLCA B & C might have targeted E6 and E7 viral oncoproteins for restoration of p53 and stabilization of pRB to induce apoptosis and G1/S phase cell cycle arrest, respectively.

Apoptosis is an important mechanism to kill the tumor cells [24] and can be induced by increase in the intracellular calcium that results into loss of membrane potential (Δψm), expansion of the matrix and rupture of the outer mitochondrial membrane [25]. This results into release of cytochrome c into the cytosol, either by inhibition of anti-apoptotic factors or activation of pro-apoptotic proteins leading to the activation of caspase 3/9. In our study, the morphological analysis by staining with Annexin V-FITC/PI demonstrated that the SLCAs-treated cells predominantly undergo apoptosis and not necrosis. Further, intracellular calcium levels and mitochondrial membrane potential studies suggest that SLCAs induced release of intracellular Ca^2+^ into the cytosol from mitochondria in HeLa cells directly or indirectly causing the mitochondrial damage followed by the mitochondrial depolarization.

Caspase activation is one of the complex and important early events in the commitment of a cell to undergo apoptosis [11]. Studies in some apoptotic models have shown the positive feedback loop of the activation of caspase-3, -8 and -9. It has also been reported that caspase-3 and -8 could activate each other downstream of the mitochondria-mediated caspase-9 activation. Moreover, the activity of caspase-9 is increased directly by activated caspase-8 and activated caspase-3, in turn, introducing more caspase-9 in a positive feedback amplification loop [13, 14]. In our study, enhanced activities of caspase-3 and -9 were observed in HeLa cells treated with SLCAs that confirmed the involvement of mitochondria-mediated apoptosis pathway. Interestingly, we observed significantly high caspase-8 activity in SLCA-treated HeLa cells, which might be due to increased active caspase-9 levels. However, caspase-9 dependent caspase-8 activation upon treatment with SLCAs needs further validation.

## Conclusions

In conclusion, this study indicates that two sophorolipids (SLCA B and SLCA C) have potential growth regulatory role in human cervical HeLa cancer cells *via* inducing apoptosis. SLCAs stimulate the activation of caspase-3, -8 and -9 with alteration of mitochondrial membrane potential. Among these two SLCAs, SLCA C was found to be more cytotoxic than SLCA B. Both SLCAs showed similar apoptotic effect in human cervical cancer cells in a time-dependent manner; arresting the cells at G1/S phase for 6, 12, 18 and 24 h. The underlying mechanism of apoptosis was result of depolarization of mitochondrial membrane and elevation in intracellular calcium level leading to activation of caspases. Thus, both SLCA B and SLCA C exhibited potential as chemopreventive anti-tumor agents that could improve common anticancer therapies.

## Supporting information

S1 Fig Ion spectra and structure of sodium adduct of non-acetylated form of cetyl alcohol sophorolipid with methyl end group. (TIF)

S2 Fig Ion spectra and structure of sodium adduct of monoacetylated form of cetyl alcohol sophorolipid with methyl end group. (TIF)

S3 Fig Graphical representation of percentage growth inhibition GI_50_ for SLCA. The data represents mean ± SD of three independent experiments.
(TIF)

## Abbreviations

A431: Epidermoid carcinoma
A549: Lung adenocarcinoma
DAPI: 6-Diamidino-2- phenylindole
FITC: Fluorescein isothiocyanate
HCS: High content screening
HCT 116: Colorectal carcinoma
HeLa: Cervical adenocarcinoma
HUVEC: Human umbilical vein endothelial cells
L929: Areolar and adipose tissue fibroblast cells
LSCM: Laser-scanning confocal microscope
MCF-7: Breast adenocarcinoma
MTT: [3-(4,5-Dimethylthiazol-2-yl)-2,5-diphenyltetrazolium bromide
PANC-1: Pancreatic carcinoma
PI: Propidium iodide
SLCA: Sophorolipid derived from cetyl alcohol
THP-1: Acute monocytic leukemia
