# Supplementary figures and images for: Anti-proliferative effect of novel primary cetyl alcohol derived sophorolipids against human cervical cancer cells HeLa
Source: PLoS One. 2017 Apr 18;12(4):e0174241. doi: 10.1371/journal.pone.0174241 (PMC5395175; doi:10.1371/journal.pone.0174241)

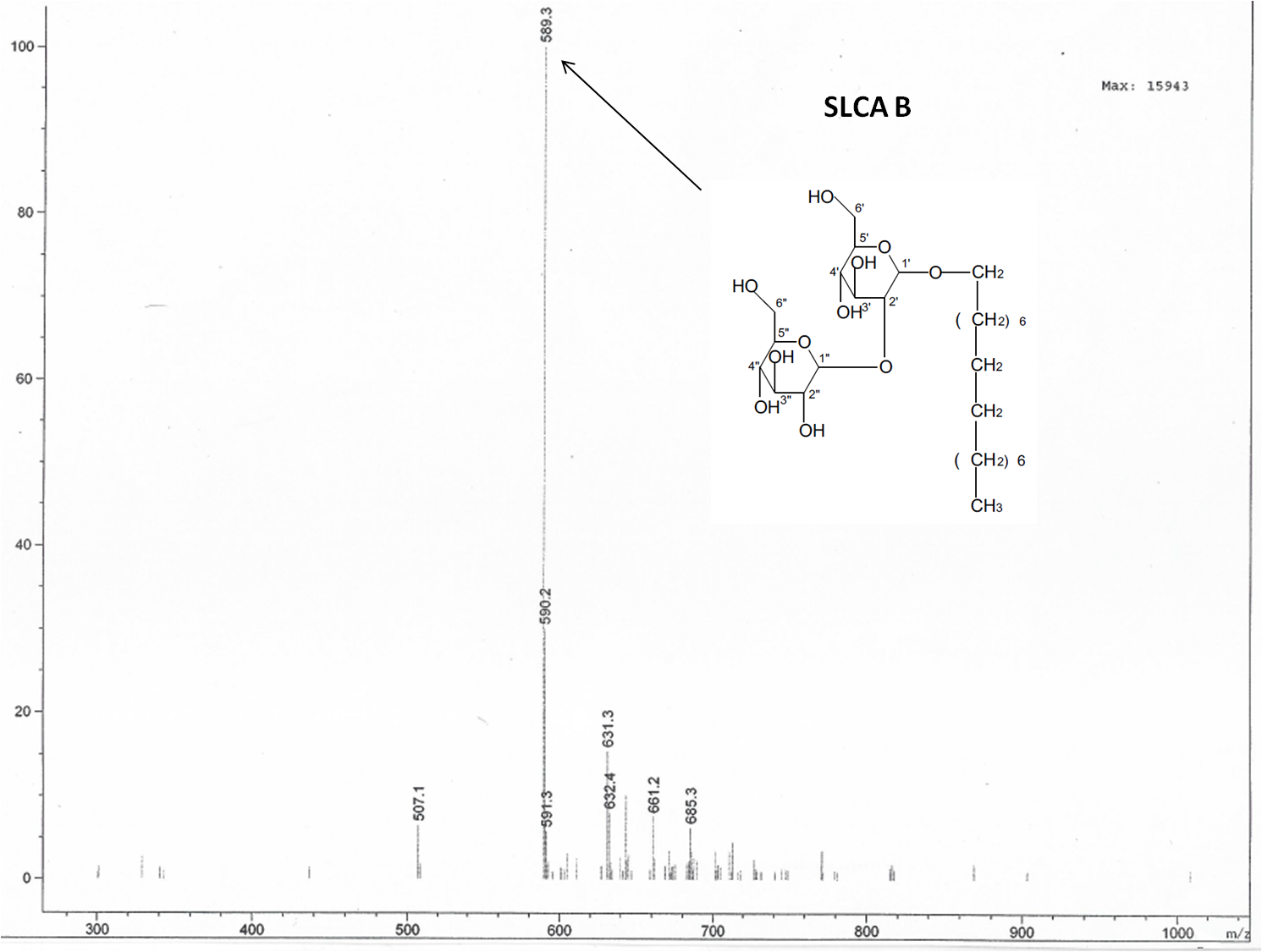

Supplement: S1 Fig — (TIF) [file pone.0174241.s001.tif]

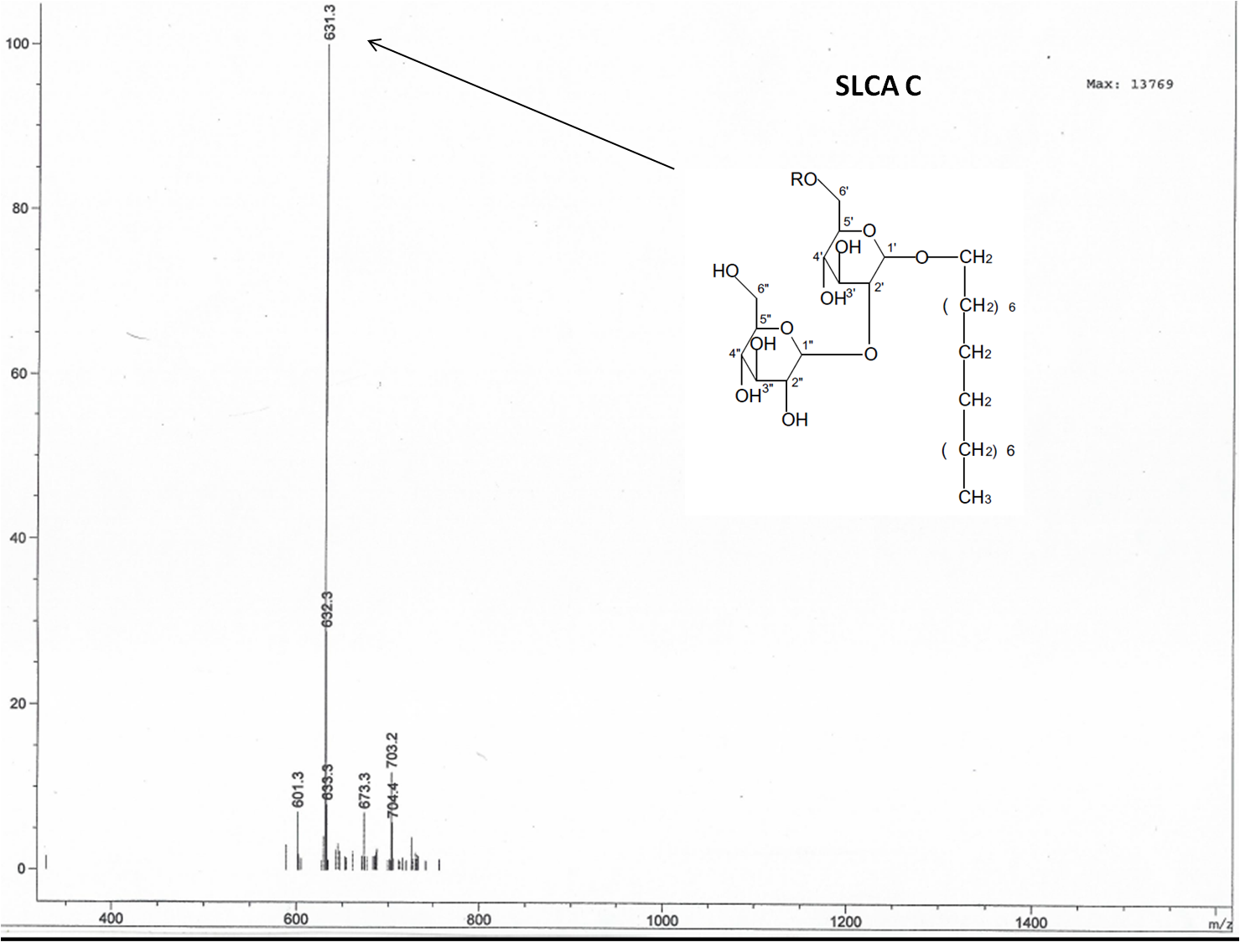

Supplement: S2 Fig — (TIF) [file pone.0174241.s002.tif]

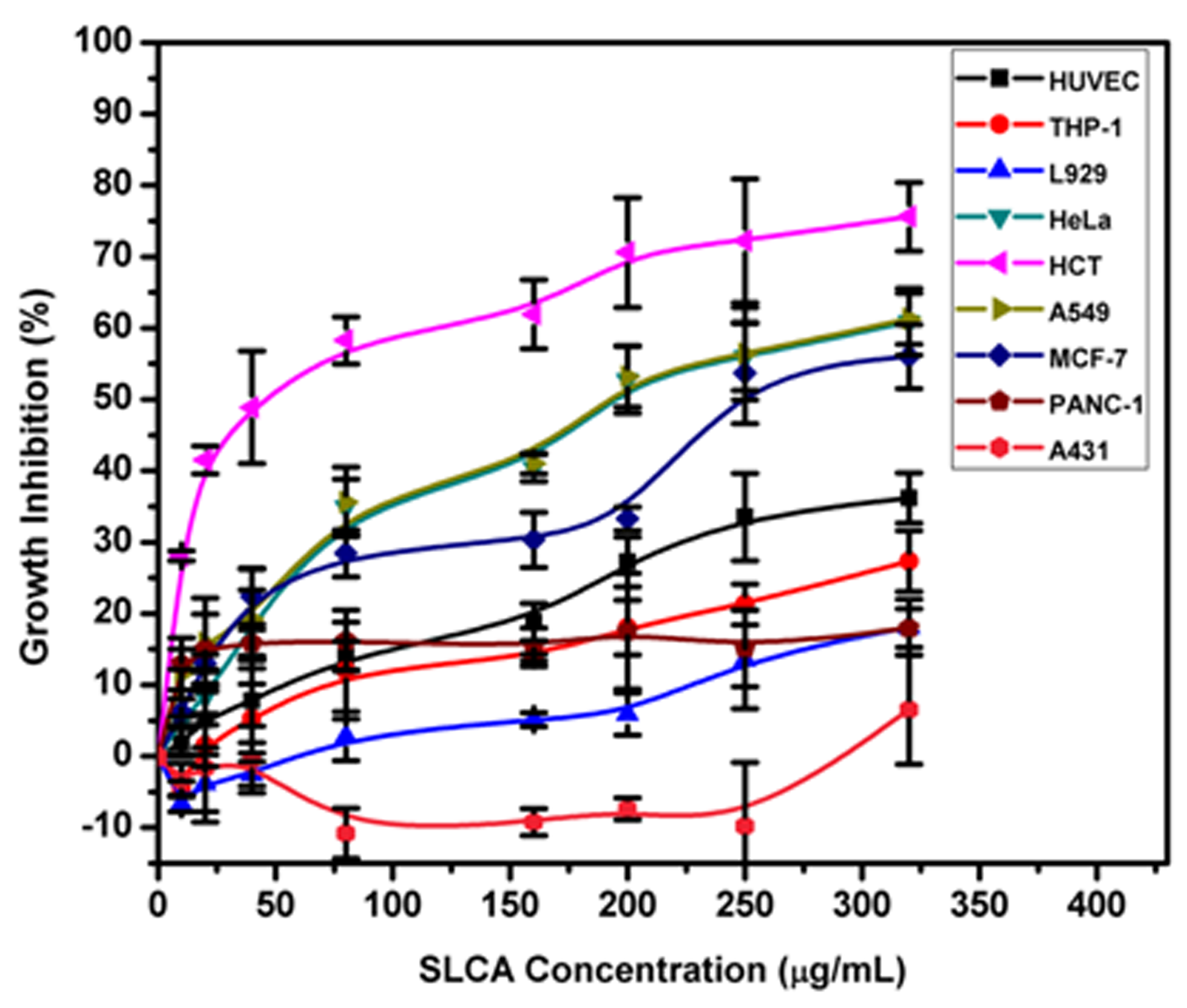

Supplement: S3 Fig — The data represents mean ± SD of three independent experiments. (TIF) [file pone.0174241.s003.tif]
